# Methods for the strategic review of programmes for integrated management of childhood illness and community cases

**DOI:** 10.1136/bmj.k2989

**Published:** 2018-07-30

**Authors:** Sarah L Dalglish

**Affiliations:** Strategic review of IMCI and iCCM, World Health Organization, Geneva, Switzerland

## Abstract

**Sarah Dalglish** describes the purpose, methods, and process used to conduct the 2016 strategic review of Integrated Management of Childhood Illness and integrated Community Case Management

**Key messages**

- The strategic review of Integrated Management of Childhood Illness and integrated Community Case Management aimed to learn from implementation of past strategies to inform recommendations on meeting global child health goals.

- Review authors synthesised 34 quantitative and qualitative data sources, with data collection methods including literature and desk reviews, in-depth interviews, a global implementation survey, and country case studies and implementation vignettes
- Data analysis was iterative and used pre-defined research questions to interrogate data, discussion at in-person analysis workshops, and consultation with global, regional, and country level stakeholders.
- As health needs, scientific evidence, and technology change, exercises such as the strategic review may help guide global and national public health strategies.

Twenty years after the introduction of Integrated Management of Childhood Illness (IMCI) as a global child health strategy, later complemented by integrated Community Case Management (iCCM), the World Health Organization and the United Nations Children’s Fund (Unicef) sought to re-envision their approach to reducing child mortality and promoting the wellbeing of children worldwide. The strategic review of IMCI and iCCM was designed to provide evidence based recommendations towards achieving child health goals under the sustainable development goals and Global Strategy for Women’s, Children’s, and Adolescents’ Health (2016-30). This article provides an overview of the strategic review’s methods to contextualise and situate the findings presented in this online collection (www.bmj.com/child-health).

In 2015, concerns about the lack of sustained interest and funding of child health globally coincided with the advent of new priorities under the sustainable development goals, prompting WHO to seek to review its global child health strategies. Initial discussion among stakeholders led to the formation of a WHO-Unicef coordinating group and an expert advisory group of independent academics for the strategic review of IMCI and iCCM. The study team formulated research questions designed to provide a comprehensive analysis of the state of the art in delivering child health services and lessons from implementation of past child health strategies, to form recommendations on how to meet current global child health goals. These topics were considered by examining 34 unique data sources, 32 specifically commissioned for the strategic review, with overlapping data collection and analysis from February to July 2016. The final dataset was both qualitative and quantitative, representing contributions from nearly 100 countries, hundreds of experts in global child health, comprehensive reviews of the scientific literature, and assessments of implementation and innovations from countries around the world (see appendix 1 for a list of data sources and further details on all methods).

## Literature and desk reviews to establish best practices in relevant technical areas

The first step in data collection was to synthesise existing knowledge on the efficacy of current child health strategies and potential areas of improvement for future strategies. Reviews of the published literature covered the following topics: previous evaluations of IMCI and iCCM, current and projected future child health epidemiology, innovations in diagnostic and treatment guidelines, communication technologies for child health, private sector initiatives, community engagement strategies, and child health in humanitarian and emergency settings. Desk reviews of unpublished data focused on adaptations to IMCI, improvement in healthcare provider performance, and WHO and Unicef internal documents on IMCI and iCCM, using documents and data identified by strategic review stakeholders at WHO, Unicef, and academic institutions. Ten literature and desk reviews were commissioned from subject area experts. Additionally, two contemporaneous non-commissioned publications were included: one on global child health leadership by the United States Agency for International Development^1^ and one on IMCI and health systems issues by Unicef.^2^

## Interviews to glean insights from experienced professionals

In-depth interviews with 20 key informants were conducted to understand successes and challenges in implementation of child health strategies and identify opportunities for improving current strategies and promoting them in the global agenda. Selection of respondents was based on their sustained, high level involvement in global child health and expanded via snowball sampling. Interviews were semi-structured and focused on respondents’ experiences with IMCI and iCCM implementation, programme management and coordination, and options for improving the delivery and management of child health strategies. Respondents had a range of experiences in global child health; over half worked in a university or research setting, and some had dual roles in research and provision of care. Interviews took place mainly by phone and were audio recorded. Analysis was performed when notes were transformed into an aide memoire for each interview^3^ and content compared across interviews, with key findings summarised in a final report.

## Global survey to provide a detailed overview of implementation

A global implementation survey was used to document the extent to which IMCI was adopted and scaled up, as well as the perspectives of implementing partners regarding challenges and successes. The survey questionnaire was developed by WHO in collaboration with Unicef, with questions on implementation and coverage of interventions, organisation and financing, development and adoption of policy and guidelines, innovations, and implementation challenges and strengths. Questionnaires were sent to 130 countries in all six WHO regions, with 95 countries retained for the final analysis. Analysis was disaggregated by WHO region, level of under 5 mortality, income category, and level of IMCI implementation using 2015 data on under-5 mortality (numbers and rates) from the UN Inter-agency Group for Child Mortality Estimation report on levels and trends in child mortality^3^ and current gross domestic product per capita (2016) using World Bank data.^4^ A single data file was created using information from all data sources and analysis was performed using STATA version 11. More detailed methods and findings are described elsewhere in this collection.^5^

## Quantitative analyses to evaluate trends and effect

Quantitative analyses were commissioned to understand the impact of IMCI and iCCM implementation at global level, and needs and service provision at country level. An overview of care seeking trends for childhood illness was commissioned to compare countries with weaker or stronger implementation of IMCI and iCCM, using data from 300 surveys in 90 countries from the Demographic and Health Surveys (DHS) programme. We used multilevel linear regression models with surveys as level one units and countries as level two units to estimate the annual percentage point change in care seeking for pneumonia and for any condition (1993-2016); these outcomes were selected as the best proxies for use of child health strategies likely to have a mortality impact in the DHS programme. Analyses were conducted at global level and by wealth quintile for each category of countries according to IMCI and iCCM implementation. Additionally, we used maps and geographical analysis to examine disease burden in under 5s and the (mis)match between needs and service provision in select focus countries where data were available (Democratic Republic of the Congo, Ethiopia, and Nigeria).^6^ We used DHS indicators on child morbidity and mortality coded with global positioning system data,^7^ along with data from the DHS’s service provision assessment survey on children’s health services and IMCI, as well as programmatic data obtained with help from in-country stakeholders. Data were used to develop maps displaying mortality, availability of health resources, and geographical distribution of programmes and interventions. 

## Country case studies to provide a detailed description of implementation challenges and successes

In-country assessments of IMCI and iCCM implementation were initially planned in the Democratic Republic of the Congo, Ethiopia, India, and Nigeria owing to their large burdens of child mortality. However, additional assessments were conducted in Bangladesh, Kazakhstan, Myanmar, Nepal, and Yemen as a result of stakeholder enthusiasm around the strategic review. These assessments aimed to identify political, policy, and health systems challenges in implementation and appraise barriers and opportunities for improving quality, access, coverage, and utilisation of child health services. Data collection and analysis tools were provided by the strategic review study team and adapted or translated in country. In most cases, an international consultant was paired with an in-country consultant or team to do a literature review and conduct interviews with child health stakeholders in the capital city and one to two outlying districts over one to two weeks. Data from interviews were entered into a structured analysis tool that allowed for comparison of respondents’ answers across predefined research questions; emerging themes were further developed via discussions between colleagues.^8^ Preliminary findings were presented to stakeholders at the ministry of health and WHO and Unicef country offices. Final reports were written in similar formats to enhance comparability across countries.

## Vignettes of successful child health interventions from around the world

The final method was more informal: we collected short vignettes of effective child health strategies from around the globe and innovations in delivering health services to children. The purpose was to provide examples of success in real world settings and spark ideas about innovations and best practices that could be extended, generalised, or built on in future policies and programmes. Eight vignettes of less than 2000 words each were collected through WHO regional offices and described activities as varied as the use of mHealth technologies to track commodity stocks in Malawi; the integration of IMCI into pre-service training in Egypt; adaptation of IMCI for emergency settings in Darfur, Sudan; and results based budgeting for child health in Peru.

## Data analysis: triangulation, iteration, consultation

Given the large amount of data considered in the strategic review, sequential analysis steps were necessary to distil findings into a set of key messages and recommendations for achieving child health goals over the next two decades. Analysis began concurrently with data collection, when study coordinators and expert advisers held regular teleconferences to discuss emerging findings and suggest adjustments to ongoing research activities. Subsequently, study team members formed groups to interrogate data sources to answer predefined research questions pertaining to IMCI and iCCM implementation, the state of the art in delivering child health interventions, and options for improving child health programmes and strategies at country and global level.

These groups presented their findings at a three day analysis workshop attended by about 15 study team members in June 2016. Given the volume of data, no participants had read all data sources, but all data sources had been read by multiple participants. Presentations and discussions enabled triangulation and reconciliation between multiple strands of data, to produce an initial list of 30 “key messages” backed up by evidence. Due consideration was given to the validity of each data source using standard criteria for data quality in quantitative and qualitative research,^9^ ^10^ and claims were weighed based on the strength of the evidence. A draft report and recommendations were written and revised internally among study group members, and then presented at a two day workshop in July 2016 with high level experts in child health, resulting in further refinements to recommendations and the final report.^11^ The articles in this collection set out and expand on the themes emerging from this report.

## Limitations and strengths of the strategic review

The strategic review sought to provide evidence based examination of past implementation, current best practices, and future actions for child health; however, limitations in data collection and analysis condition evaluation of its findings. The strategic review was completed on an accelerated timeline to accommodate donor funding cycles, and there was insufficient exploration of some of the research commissioned—for example, in child health in emergency and humanitarian settings. This omission has been accounted for in subsequent work, including a WHO research prioritisation process on child health in humanitarian settings and in the forthcoming WHO/Unicef Lancet commission on child wellbeing and health. Findings were also limited by a lack of input from beneficiaries, including children’s mothers and families, because of limited time to engage in consultation processes. Future reviews in this vein might benefit from more systematic planning, codified involvement of stakeholders at multiple levels, and the establishment of a predefined consultation process to increase confidence in findings.

Additional limitations are specific to data collection methods. Global key informant interviews reached only a few of the relevant informants, and because of time restrictions, interviews were not transcribed verbatim. Thus thematic analysis may have focused on pre-established themes to the exclusion of those emerging from the data. Country assessments were performed rapidly, and there was probably bias in selecting evaluation districts because most remote districts were less likely to be included; however, geographical inequity was explored using other methods (notably global positioning system/geographical information system mappings). Initially unplanned assessments were conducted in Bangladesh, Kazakhstan, Myanmar, Nepal, and Yemen at the request of enthusiastic local stakeholders, which may have provided a positively biased sample (although many implementation challenges were noted in these settings). The IMCI survey data had limitations common to survey design and which are detailed elsewhere in this collection.^5^

The strategic review was retrospective, and many data collection methods called on people involved in designing and implementing the strategies in question to evaluate their appropriateness, effectiveness, strengths, and flaws. Evaluating potential subjectivities based on respondents’ positions within child health organisations was not always easy. In other cases, those interviewed for the strategic review were newly appointed and not able to draw on institutional memory about IMCI and iCCM. Thus both respondent and recall bias are potential risks. To mitigate for these risks, members of the analysis teams in countries and at global level were selected for their long term engagement with the issues examined, providing historical perspective and context to a short term, retrospective review.

The principal strengths of the strategic review are its broad and deep dataset and its iterative analysis, allowing for the extraction of overarching, high level messages. To reach their recommendations, authors of the strategic review analysed nearly 1000 pages of original data over the course of multiple sessions using diverse data analysis methods. This unique process was designed to allow a full reckoning with some of the most challenging questions in global public health today. In addition to sharing our findings, we hope to spark discussion about our process, including the usefulness of such an exercise for considering evolving global public health strategies, such as those protecting the health of children.

## Web Extra

Extra material supplied by the author

Appendix: Methods and data sources for strategic review

## Notes

See www.bmj.com/child-health for other articles in the series.

## Notes

Contributors and sources: SD worked with the principal investigator (Anthony Costello) and members of the study team to design and implement the strategic review. The study protocol was designed by members of the coordinating group (Samira Aboubaker, Rajiv Bahl, Cynthia Boschi-Pinto, Berandette Daelmans, Theresa Diaz, Nicholas Oliphant, Jonathon Simon, Joanna Vogel, Wilson Were, Mark Young) and members of the expert advisory group (Tim Colbourn, Tanya Doherty, Youssouf Gamatié, Rasa Izadnegahdar, Elizabeth Mason, Smruti Patel, Alexander Rowe, Eric Simoes). These groups were also responsible for data analysis and for the final report of the strategic review.

Provenance and peer review: Commissioned; externally peer reviewed.

This article is part of a series based on findings from a global review of Integrated Management of Childhood Illness funded by the Bill and Melinda Gates Foundation. Open access fees were funded by the Health Systems Research Unit, South African Medical Research Council. *The BMJ* peer reviewed, edited, and made the decision to publish the article with no involvement from the Medical Research Council.
