# Supplementary material for: Methods for the strategic review of programmes for integrated management of childhood illness and community cases
Source: BMJ. 2018 Jul 30;362:k2989. doi: 10.1136/bmj.k2989 (PMC6063343; doi:10.1136/bmj.k2989)
Supplement: Supplementary file 1 — Appendix: Methods and data sources for strategic review [file dals40542.ww1.pdf]

Appendix 1: Methodology & sources of data for the Strategic Review

This appendix presents additional information on the methodology and data sources of the 2016 WHO/UNICEF Strategic Review of IMCI and iCCM. These data sources, in addition to the overall research protocol for the review, can be accessed by clicking here.

Literature & desk reviews

1. "IMCI evolution and timeline"
   Cathy Wolfheim, Bernadette Daelmans, Samira Aboubaker
2. "Integrated Management of Childhood Illness (IMCI) in the 21st Century: Present situational analysis, integration into health systems and innovations" (UNICEF working paper)
   Igor Rudan, Smruti Patel, Donald Waters, Kerri Wazny, Iain Campbell, Devi Sridhar, Mickey Chopra and Harry Campbell
3. "Review of the Literature for the Strategic Review (published & unpublished)" (Excel file)
   Guilhem Labadie
4. "Adaptations to IMCI 2006-2016"
   Eva Kudlova
5. "Innovations in the World Health Organization diagnostic and treatment guidelines for newborns and children"
   W. Chris Buck, Nichola Connell, Michelle Eckerle, Azadeh Farzin, Amy Ginsburg, Carlos Grijalva-Eternod, Matthew S. Kelly, Marko Kerac, Marie McGrath, Rashmi Patil, Michele Usuelli, and Eric D. McCollum
6. "Health Care Provider Performance Review: Identifying strategies to improve health worker performance, increase utilization of health services, and reduce mortality in LMIC"
   Guilhem Labadie with Alexander Rowe
7. "Information and Communication Technologies to Advance Child Health"
   Smisha Agarwal and Alain B. Labrique
8. "Engaging the Private Sector for Delivery of Child Health Interventions: The role of IMCI"
   Phyllis Awor
9. "Predictions/recommendations for future IMCI taking into account mortality reductions and changing causes of death as well as etiologies within the major killers"
   Eric Simoes and Sandy Gove
10. "Building on Community-IMCI: Community approaches that strengthen the capabilities of individuals, families and communities to improve child health in high-mortality settings"
    Audrey Prost
11. "Tools and Strategies for Child Health and Survival in Humanitarian Emergencies: Current Practices and Needs"
    Simran Chaudhri
12. "Mapping Global Leadership in Child Health" (USAID/MCSP report)

Mary E. Taylor, Renata Schumacher, and Nicole Davis

Global key informant interviews

13. "Global key informant interviews for the Strategic Review – Synthesis report"
    Jennifer Franz-Vasdeki

Global survey of IMCI and iCCM implementation

14. IMCI Global Implementation Survey Report
    Cynthia Boschi-Pinto, Dilip Thandassery, Samira Aboubaker, Wilson Were, Bernadette Daelmans, Eva Kudlova

Quantitative analyses of DHS and other sources

15. "Global trends in care-seeking by IMCI and iCCM implementation"
    Cesar Victora and Aluisio Barros
16. "Mapping and GIS Analysis of IMCI programming and child health outcomes: Ethiopia, Nigeria and the DRC"
    Elisabeth Root and Eric Simoes
17. "Analysis of DHS data on care-seeking: Ethiopia, Nigeria, DRC, India"
    Saverio Bellizzi

Country assessments

18. Bangladesh
    Ashok Patwari, Tahmina Begum and Rabeya Khatoon
19. Democratic Republic of the Congo
    Youssouf Gamatié, Celestin Nsibu and Brigitte Kini
20. Ethiopia
    Samira Aboubaker, Abiy Seifu Estifanos and Sarah Dalglish
21. India
    Sutapa B Neogi, Monika Chauhan
22. Kazakhstan
    Aigul Kuttumuratova, Gaukhar Abuova
23. Myanmar
    Elizabeth Mason, Thwe Thwe Win, Anoma Jayathilaka
24. Nepal
    Ram Bhandari, Benu Bahadur Karki, Jyoti Ratna Dhakwa, Bijeta Bhandari, Eric Simoes
25. Nigeria
    Andrew Mbewe, Robinson Wammanda and Sarah Dalglish
26. Yemen
    Huda Al-Naggar

Vignettes of successful interventions

27. Burkina Faso: "Story of REC" (electronic tablet tool)
    Olga Agbodjan-Prince, Fousséni Dao, Noël Adannou Zonon
28. Egypt: "A systematic approach for implementing IMCI"
    Suzanne Farhoud
29. WHO Eastern Mediterranean Region: "IMCI pre-service education: a unique experience"
    Suzanne Farhoud

30. Kyrgyzstan: “Implementation of the Pocket book led to improving quality of care for children and reducing unnecessary hospitalizations”
    Venera Shukurova
31. Malawi: “Sustaining Our Investments in cStock: Sustaining Gains in Child Health”
    Humphreys Nsona, Leslie Mgalula and Amos Misomali
32. Pakistan: “Sehat Ki Dastak: Health Knocking at the Door”
    Durdana Poonam
33. Peru: “Results-based budgeting programmes for maternal and child health”
    Luis Huicho and Jessica Niño de Guzman
34. Sudan: “Scaling of IMCI Interventions During Emergency: A case study from Darfur”
    Sumaia Mohamed Alfadil
